# Superoxide Dismutase Mimetic GC4419 Enhances the Oxidation of Pharmacological Ascorbate and Its Anticancer Effects in an H_2_O_2_-Dependent Manner

**DOI:** 10.3390/antiox7010018

**Published:** 2018-01-19

**Authors:** Collin D. Heer, Andrew B. Davis, David B. Riffe, Brett A. Wagner, Kelly C. Falls, Bryan G. Allen, Garry R. Buettner, Robert A. Beardsley, Dennis P. Riley, Douglas R. Spitz

**Affiliations:** 1 Free Radical and Radiation Biology Program, Department of Radiation Oncology, Holden Comprehensive Cancer Center, The University of Iowa College of Medicine, Iowa City, IA 52242, USA; andrew-davis-2@uiowa.edu (A.B.D.); david-riffe@uiowa.edu (D.B.R.); brett-wagner@uiowa.edu (B.A.W.); kelly-falls@uiowa.edu (K.C.F.); bryan-allen@uiowa.edu (B.G.A.); garry-buettner@uiowa.edu (G.R.B.); 2 Galera Therapeutics, Malvern, PA 19355, USA; rbeardsley@galeratx.com (R.A.B.); driley@galeratx.com (D.P.R.)

**Keywords:** SOD mimetic, pharmacological ascorbate, vitamin C, head and neck cancer, lung cancer, radiation therapy, GC4419, oxidative stress, hydrogen peroxide

## Abstract

Lung cancer, together with head and neck cancer, accounts for more than one-fourth of cancer deaths worldwide. New, non-toxic therapeutic approaches are needed. High-dose IV vitamin C (aka, pharmacological ascorbate; P-AscH^−^) represents a promising adjuvant to radiochemotherapy that exerts its anti-cancer effects via metal-catalyzed oxidation to form H_2_O_2_. Mn(III)-porphyrins possessing superoxide dismutase (SOD) mimetic activity have been shown to increase the rate of oxidation of AscH^−^, enhancing the anti-tumor effects of AscH^−^ in several cancer types. The current study demonstrates that the Mn(II)-containing pentaazamacrocyclic selective SOD mimetic GC4419 may serve as an AscH^−^/O_2_^•−^ oxidoreductase as evidenced by the increased rate of oxygen consumption, steady-state concentrations of ascorbate radical, and H_2_O_2_ production in complete cell culture media. GC4419, but not CuZnSOD, was shown to significantly enhance the toxicity of AscH^−^ in H1299, SCC25, SQ20B, and Cal27 cancer cell lines. This enhanced cancer cell killing was dependent upon the catalytic activity of the SOD mimetic and the generation of H_2_O_2_, as determined using conditional overexpression of catalase in H1299T cells. GC4419 combined with AscH^−^ was also capable of enhancing radiation-induced cancer cell killing. Currently, AscH^−^ and GC4419 are each being tested separately in clinical trials in combination with radiation therapy. Data presented here support the hypothesis that the combination of GC4419 and AscH^−^ may provide an effective means by which to further enhance radiation therapy responses.

## 1. Introduction

Lung cancer, along with head and neck cancer, accounts for more than one-fourth of cancer deaths worldwide [1]. New, non-toxic therapeutic approaches are needed to improve survival outcomes. An emerging adjuvant therapy currently in clinical trials is high-dose IV vitamin C (aka, pharmacological ascorbate, P-AscH^−^). Pharmacological ascorbate has shown promise when used in combination with radiation and chemotherapy in glioblastoma multiforme (NCT02344355), lung cancer (NCT02420314), and pancreatic cancer (NCT02905578) [2,3,4,5]. At physiological concentrations (40–80 μM in blood plasma), AscH^−^ serves as a reducing agent and donor antioxidant [6]. However, at sufficiently high plasma concentrations (mM), AscH^−^ can act as a selective pro-oxidant in cancer cells [2,5,7]. The pro-oxidant effects of AscH^−^ are significantly increased in the presence of redox-active transition metals. With a metal catalyst, AscH^−^ can rapidly transfer an electron to oxygen yielding O_2_^•−^. The O_2_^•−^ is then dismuted to H_2_O_2_ spontaneously or by the superoxide dismutase (SOD) family of enzymes [7,8]. The toxicity of AscH^−^ to cancer cells can be inhibited by either catalase, which removes H_2_O_2_, or iron chelators that inhibit redox cycling. Thus, Fenton chemistry involving H_2_O_2_ and labile iron is thought to be responsible for the cancer-cell-specific cytotoxic effects of AscH^−^ in vitro [5,9]. The role of transition metals in AscH^−^ pro-oxidant chemistry has roused interest in combining AscH^−^ with pharmaceutical agents containing redox-active transition metals. For example, the manganese–porphyrin [Mn(III)-porphyrin] class, which has SOD mimetic activity, has been shown to enhance AscH^−^ toxicity toward cancer cells by increasing the generation of H_2_O_2_ in breast and pancreatic cancers [10,11,12,13,14].

A prominent class of SOD mimetics is the pentaazamacrocyclic Mn(II)-containing compounds, such as GC4419. GC4419 has exhibited therapeutic effects in several animal models and has recently completed a multi-site phase 2b clinical trial that investigated its ability to mitigate oral mucositis, a commonly experienced side effect of chemo-radiation and radiation therapy in head and neck cancer (NCT02508389) [15,16,17]. GC4419 is highly stable at pH 7.4 and selectively catalyzes the dismutation of O_2_^•−^ with a rate constant at 2 × 10^7^ M^−1^ s^−1^ [18]. GC4419 has been shown to selectively protect normal, but not malignant, human cells in culture and normal human tissue in a Phase 1b/2a clinical study, from radiation and chemotherapy [19,20]. 

While some Mn(III)-porphyrin SOD mimetics have been shown to augment the redox cycling and subsequent toxicity of AscH^−^, the ability of Mn(II)-containing SOD mimetics to enhance the anticancer effects of AscH^−^ has not been thoroughly investigated, primarily because the resting state of the pentaazamacrocyclic compounds is Mn(II) and is thus not amenable to reduction by AscH^−^. However, it has been suggested that M40403 (aka, GC4403), a Mn(II)-pentaazamacrocyclic compound that is the mirror-image isomer of GC4419, may function as a AscH^−^/O_2_^•−^ oxidoreductase that catalyzes the transfer of an electron from AscH^−^ to O_2_^•−^, yielding H_2_O_2_ and Asc^•−^ [21]. Yet, the ability of GC4419 to enhance the anticancer effects of AscH^−^ remains unknown. Here we present evidence that, when combined with pharmacological levels of AscH^−^, GC4419 significantly increases cancer cell killing relative to AscH^−^ alone. Our data suggest this cancer cell killing is dependent on H_2_O_2_ and is accompanied by increased rates of oxidation of AscH^−^, oxygen consumption, and H_2_O_2_ production. In addition, GC4419 + AscH^−^ (GC/AscH^−^) sensitizes human lung and head and neck cancer cells to radiation. Both AscH^−^ and GC4419 are currently being investigated individually in phase 2 clinical trials. Because both of these trials involve radiation therapy, the data presented here support the hypothesis that the combination of these agents may represent a viable approach to significantly improve patient outcomes in both head and neck and lung cancer. 

## 2. Materials and Methods 

### 2.1. Cell Lines and Media

H1299 lung cancer cells were obtained from ATCC and grown in RPMI 1640 media supplemented with 10% fetal bovine serum (FBS; Atlanta Biologicals, Flowery Brach, GA, USA). Cal27 and SCC25 head and neck cancer cells were obtained from ATCC. SQ20B cells were a gift from Andrean Simons-Burnett (Department of Pathology, University of Iowa, Iowa City, IA, USA). TSA201 cells were a gift from Dawn Quelle (Department of Pharmacology, University of Iowa, Iowa City, IA, USA). Cal27, SQ20B and TSA201 cells were maintained in DMEM (Gibco, Grand Island, NY, USA) media supplemented with 10% FBS. SCC25 cells were maintained in DMEM:F12 (Gibco) media containing 10% FBS, 1% HEPES and 400 ng mL^−1^ hydrocortisone. H1299T-CAT cells were derived from the H1299T cell line, as described below. All cultures were maintained in 5% CO_2_, 20% O_2_, and humidified in a 37 °C incubator. 

### 2.2. Drug Treatment

Cells were treated with 5–20 μM GC4419 for 1 h or 24 h prior to and during treatment with AscH^−^ (2.5–20 pmol cell^−1^). l-Ascorbic acid stock solution (1.0 M) was prepared in Nanopure Type 1 water with the pH adjusted to 7.0 with NaOH. Concentration was determined spectrophotometrically, ε_265_ = 14,500 M^−1^ cm^−1^ [8]. AscH^−^ was dosed per cell (pmol cell^−1^), rather than by concentration, due to the dependence of AscH^−^ and H_2_O_2_ toxicity on cell density [5,22,23,24]. All concentrations used in this study are relevant to in vivo measurements of AscH^−^ in human plasma following IV infusion. GC4419 was dissolved in Nanopure water containing 5 mM sodium bicarbonate (Gibco). CuZnSOD (DDI Pharmaceuticals, Mountain View, CA, USA) was dissolved in Nanopure water. Cells were irradiated with a dose of 2 Gy (dose rate, 0.365 Gy min^−1^) using a ^37^Cs source (JL Shepherd, San Fernando, CA, USA). 

### 2.3. Electron Paramagnetic Resonance (EPR) Spectroscopy 

Spectra of the ascorbate free radical were collected with a Bruker EMX ESR spectrophotometer (Bruker BioSpin, Billerica, MA, USA) as previously described [10]. EPR instrument settings used to quantify [Asc^•−^] were center field, 3507.62 G; sweep width, 10.00 G; receiver gain, 5.02 × 10^4^; modulation amplitude, 0.70 G; microwave frequency, 9.85 GHz; using an ER4119HS cavity with nominal microwave power of 10.0 mW. 3-Carboxyl-PROXYL (3-CxP, CAS: 2154-68-9; Sigma Aldrich, St. Louis, MO, USA) was used as the concentration standard taking into account potential saturation effects [25]. 

### 2.4. Oxygen Consumption by Clark Electrode 

The rate of oxygen consumption was determined using a Clark electrode (YSI Inc., Yellow Springs, OH, USA). Data were collected using an ESA Biostat multielectrode system (ESA Dionex Corp, Chelmsford, MA, USA) and analyzed using Microsoft Excel (Microsoft, Redmond, WA, USA). Rates were determined from oxygen concentrations recorded every second over at least 60 s. Experiments were carried out in RPMI media containing 10% FBS. 

### 2.5. H_2_O_2_ Quantification 

Accumulation of H_2_O_2_ was determined using a Clark electrode (YSI Inc., Yellow Springs, OH, USA). AscH^−^, GC4419 + AscH^−^, or CuZnSOD + AscH^−^ were added to RPMI media containing 10% FBS and O_2_ concentrations were recorded every second. After 10 min, the concentration of H_2_O_2_ was determined based on the amount of O_2_ generated following addition of 500 U mL^−1^ of catalase. The rate of production of H_2_O_2_ was then determined in units of nM H_2_O_2_ s^−1^. 

### 2.6. SOD Activity Assay

SOD activity was determined using the indirect competitive inhibition assay as described previously [26]. Briefly, the flux of superoxide generated by the metabolism of xanthine by xanthine oxidase was measured using nitroblue tetrazolium (NBT). The rate of NBT reduction can be measured spectrophotometrically at 560 nm and is inhibited by increasing concentrations of SOD or GC4419 that compete for the superoxide produced by xanthine oxidase. The rate of NBT reduction is calculated as percent inhibition relative to the NBT reduction in the absence of the native CuZnSOD or GC4419. The amount of CuZnSOD or GC4419 causing 50% maximum inhibition is defined as one unit of activity.

### 2.7. Clonogenic Assay

Cells (1.5–3.5 × 10^5^) were plated in 60-mm dishes and allowed to grow for 48 h (SCC25) or 72 h (H1299, Cal27, SQ20B) prior to clonogenic assay. Cells were detached using 0.25% trypsin, combined with floating cells, and pelleted via centrifugation at 335× *g* for 5 min. Pellets were resuspended in fresh media and the total cell population was counted using a Beckman Coulter Counter (Beckman Coulter Inc., Hialeaha, FL, USA). Cells were then plated in 60-mm dishes at a variety of densities ranging from 150–100,000 cells per dish. Clones were grown for 7–14 days in complete media with 0.1% gentamycin, fixed with 70% ethanol, stained with Coomassie blue, and colonies containing ≥50 cells were counted. The plating efficiencies of treatment groups for each cell line were normalized to the Control, GC4419, or GC4419/doxycycline (GC/Dox)-treated groups. The survival analysis was performed using a minimum of two cloning dishes per experimental condition, and the experiments were repeated a minimum of three times. 

### 2.8. Lentivirus Production and Transduction 

The doxycycline-inducible catalase overexpression plasmid was generated as previously described and obtained from Fenghuang Zahn’s Lab (Department of Internal Medicine, University of Iowa, Iowa City, IA, USA) [27]. Lentivirus was produced in the TSA201 cell line using pCMV-VSV-G and psPAX2 helper vectors (Addgene, Caimbridge, MA, USA). H1299T cells (a clonal population derived from H1299 for aggressive growth in animals) were plated and allowed to grow for 48 h, and then virus was added to cells with 8 µg mL^−1^ of polybrene every 24 h for two days. After transduction, cells were selected with 5 µg mL^−1^ puromycin. Surviving cells were plated in 150-mm dishes with 1000 cells per dish. Clones were grown for 10 days, and then several colonies were picked and expanded. To test for maximal catalase overexpression, cells were treated with 1.5 µg mL^−1^ of doxycycline for 48 h. Protein concentration was determined using the Lowry Assay. Increased catalase activity was verified by measuring the decomposition of H_2_O_2_ by cell lysates as previously described [28]. Maximal activity was found in clone 15. Optimal dose of doxycycline was determined by treating with 0.25–2 µg mL^−1^ doxycycline for 24 or 48 h. 

### 2.9. Statistical Analysis

Statistical analyses were performed using GraphPad Prism (Graphpad Software Inc., La Jolla, CA, USA) and an α = 0.05. Two-way ANOVA analyses were performed with Tukey’s multiple comparison post hoc test. 

## 3. Results 

### 3.1. GC4419 Increases the Rate of Oxygen Consumption and Ascorbate Radical Steady-State Concentration in Systems Containing AscH^−^

**Figure 1:**
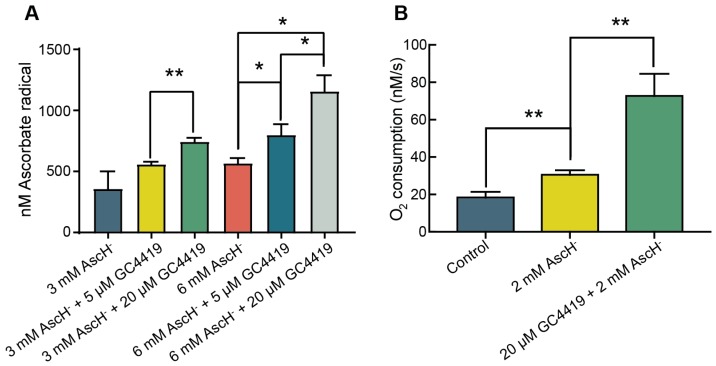
GC4419 increases the oxidative flux of systems containing AscH^−^ (**A**) GC4419 increases [Asc^•−^]_ss_ in complete media containing AscH^−^. Ascorbate alone or GC/AscH^−^ were added to complete RPMI media. Solutions were immediately examined by EPR and [Asc^•−^]_ss_ was determined. 3-Carboxyl-PROXYL was used as a concentration standard [25]. Errors represent ± SEM, *n* = 3. * *p* < 0.05, ** *p* < 0.01 by unpaired *t*-test. (**B**) GC4419 increases oxygen consumption of complete media containing AscH^−^. Ascorbate was added to complete RPMI media (Control) and the rate of oxygen consumption was measured. GC4419 was then added to the solution and oxygen consumption was measured. Errors represent ± SEM, *n* = 3. ** *p* < 0.01 by unpaired *t*-test.

To determine the effect of GC4419 on the rate of AscH^−^ oxidation in complete RPMI media, we measured the steady-state concentration of ascorbate radical ([Asc^•−^]_ss_) by EPR spectroscopy [29]. After adding AscH^−^ or GC/AscH^−^ to complete media, the solution was vortexed and immediately examined by EPR spectroscopy. When combined with 6 mM AscH^−^, addition of 5 μM or 20 μM GC4419 resulted in a dose-dependent increase in [Asc^•−^]_ss_ compared to AscH^−^ alone (Figure 1A). When added to 6 mM AscH^−^, 5 μM GC4419 increased the [Asc^•−^]_ss_ by approximately 50%. When added to 3 mM or 6 mM AscH^−^, 20 μM GC4419 roughly doubled the [Asc^•−^]_ss_. These findings support the hypothesis that GC4419 catalyzes the oxidation of AscH^−^. 

To determine if the increase in [Asc^•−^]_ss_ in the presence of GC4419 was accompanied by an increase in the production of reactive oxygen species (ROS), we evaluated the rate of oxygen consumption using a Clark Electrode. We observed that addition of 2 mM AscH^−^ to complete RPMI media resulted in an increase in the rate of O_2_ consumption from 15 to 35 nM s^−1^. The addition of 20 μM GC4419 doubled the rate of O_2_ consumption to 70 nM s^−1^ (Figure 1B). These findings support the hypothesis that the addition of GC4419 to solutions containing AscH^−^ significantly increases the rate of oxygen consumption, which may indicate the production of ROS. 

### 3.2. GC4419, but Not CuZnSOD, Increases H_2_O_2_ Production and Cancer Cell Toxicity when Combined with AscH^−^

**Figure 2:**
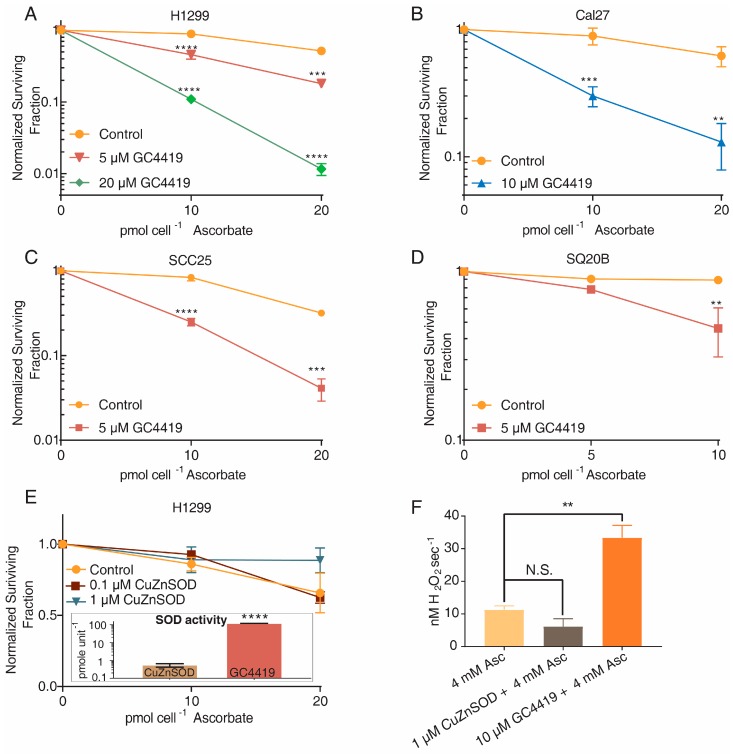
GC4419, but not CuZnSOD, increases cancer cell toxicity and H_2_O_2_ production when combined with AscH^−^. (**A**–**D**) When added 24 h prior to and during 1 h AscH^−^ treatment, GC4419 significantly increases the toxicity of AscH^−^ in lung (H1299) and head-and-neck (Cal27, SQ20B, SCC25) cancer cell lines. Errors represent ± SEM, *n* = 3 with at least two cloning dishes per treatment. ** *p* < 0.01, *** *p* < 0.001, **** *p* < 0.0001 vs. Control by two-way ANOVA. Normalized to Control or GC4419-treated group. (**E**) Cells were treated with 0.1–10 μM CuZnSOD for 24 h prior to and during AscH^−^ treatment. *n* = 4 with at least two cloning dishes per treatment. Normalized to Control or CuZnSOD groups. Inset: CuZnSOD exhibits approximately 240 times greater specific SOD activity relative to GC4419 as determined using a NBT-based competitive inhibition assay. Errors represent ± SEM, *n* = 3. **** *p* < 0.0001 vs. CuZnSOD by upaired *t*-test (**F**) Accumulation of H_2_O_2_ was determined using a Clark electrode (YSI Inc., Yellow Springs, OH, USA). AscH^−^, GC4419 + AscH^−^ or CuZnSOD + AscH^−^ were added to RPMI media containing 10% FBS. The concentration of H_2_O_2_ was determined based on the amount of O_2_ generated following addition of 500 U mL^−1^ of catalase. Errors represent ± SEM, *n* = 3–4. ** *p* < 0.01, vs. Control by unpaired *t*-test.

To determine if the increased rate of oxygen consumption and higher [Asc^•−^]_ss_ of systems containing GC4419 and AscH^−^ translates to increased cancer cell killing, cancer cells were treated with 5–20 μM GC4419 for 24 h prior to and during the addition of AscH^−^, then clonogenic survival was assessed. In multiple cancer cell lines, GC4419 significantly increased clonogenic cancer cell death in a manner that was dependent on the concentration of both GC4419 and AscH^−^ (Figure 2A–D).

We next hypothesized that the increased oxygen consumption, [Asc^•−^]_ss_, and cancer cell killing upon addition of GC4419 to systems containing AscH^−^ may be due to the SOD activity of the mimetic. Specifically, GC4419 could enhance the rate of dismutation of O_2_^•−^ produced by AscH^−^, increasing the flux of H_2_O_2_ and resulting toxicity toward cancer cells. To test this hypothesis, H1299 lung cancer cells were treated with 0.1 or 1 μM CuZnSOD 24 h prior to and during a 1 h exposure to AscH^−^. Concentrations of 0.1 and 1.0 μM CuZnSOD were chosen because these concentrations should be kinetically equivalent to 10 and 100 μM GC4419 based on rate constants for the dismutation reaction, 1.3 × 10^9^ M^−1^ s^−1^ for CuZnSOD [30] and 2 × 10^7^ M^−1^ s^−1^ for GC4419 [18]. The higher SOD activity of native CuZnSOD relative to GC4419 was confirmed using a NBT-reduction-based competitive inhibition assay where we found that the specific activity of CuZnSOD is approximately 240 times greater than that of GC4419 (Figure 2E, Inset). Interestingly, despite its higher SOD activity, no enhancement of cancer cell toxicity was observed upon addition of CuZnSOD to media containing AscH^−^ (Figure 2E). 

To determine if H_2_O_2_ production was increased upon the addition of GC4419, but not CuZnSOD, to systems containing AscH^−^, we measured the generation of O_2_ upon the addition of catalase to systems containing AscH^−^ alone, GC4419 and AscH^−^, or CuZnSOD and AscH^−^. We found that GC4419, but not CuZnSOD, was able to significantly increase accumulation of H_2_O_2_ in systems containing AscH^−^ (Figure 2F). These results suggest that increasing the SOD activity of the system does not affect H_2_O_2_ production or AscH^−^ toxicity toward cancer cells. Similar results have been observed by other researchers combining PEG-SOD with AscH^−^ [10]. These results also suggest that the SOD activity of GC4419 is not directly responsible for its ability to enhance AscH^−^ toxicity, and support the hypothesis that GC4419 functions as an AscH^−^/O_2_^•−^ oxidoreductase that catalyzes the transfer of an electron from AscH^−^ to O_2_^•−^ to produce H_2_O_2_.

### 3.3. GC/AscH^−^ Toxicity is Dependent on H_2_O_2_

**Figure 3:**
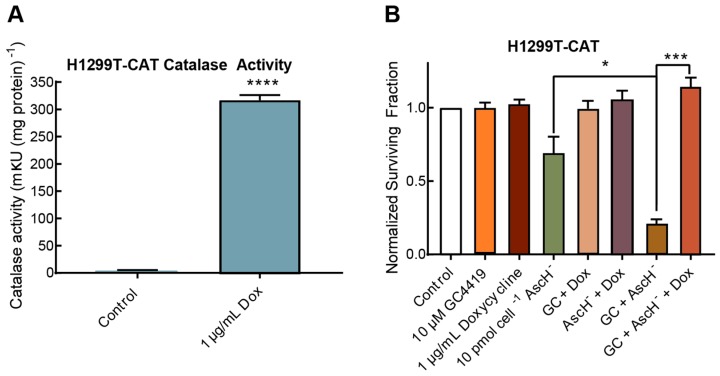
Anticancer effect of GC/AscH^−^ is dependent on H_2_O_2_. (**A**) Catalase overexpression was verified in H1299T-CAT lung cancer cell lines exposed to 1 μg mL^−1^ doxycycline. Errors represent ± SEM, *n* = 12. **** *p* < 0.0001 vs. Control by unpaired *t*-test. (**B**) Overexpression of catalase completely rescued H1299T-CAT cells from toxicity induced by GC/AscH^−^. Errors represent ± SEM, *n* = 3 with at least two cloning dishes per treatment. * *p* < 0.05, *** *p* < 0.001 by unpaired *t*-test. Normalized to Control.

To verify that the anti-cancer effect of GC/AscH^−^ treatment is H_2_O_2_-dependent, we utilized an H1299T cell line that conditionally overexpresses catalase when exposed to doxycycline (H1299T-CAT). Cells were treated with 1 μg mL^−1^ of doxycycline 48 h prior to treatment with GC4419 and AscH^−^. Doxycycline treatment was confirmed to increase catalase activity from 3.7 ± 1.5 m*k*U (mg protein)^−1^ to 316 ± 9 m*k*U (mg protein)^−1^ (Figure 3A). The cancer cell clonogenic killing capacity of GC/AscH^−^ was completely inhibited by catalase overexpression, demonstrating that H_2_O_2_ was causally related to the cancer cell clonogenic killing (Figure 3B).

### 3.4. Catalytic Activity of Mn(II)-Pentaazamacrocylces is Required for Enhancement of AscH^−^ Toxicity 

**Figure 4:**
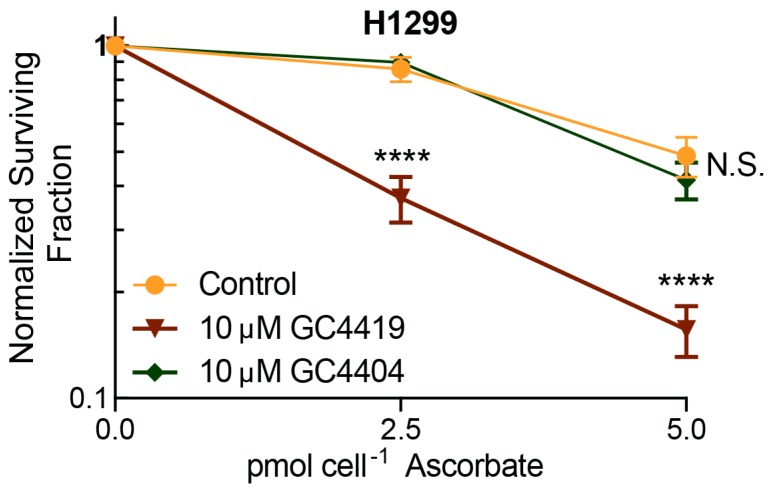
Catalytic activity of pentaazamacrocyclic mimetic is required to enhance AscH^−^ toxicity as seen by clonogenic assay. Media were replaced and cells were treated with 10 μM of GC4419 or GC4404 (SOD inactive) for 1 h prior to 1 h AscH^−^ treatment. Errors represent ± SEM, *n* = 4–7 with at least two cloning dishes per treatment. **** *p* < 0.0001, N.S. = no significance vs. Control by two-way ANOVA. Normalized to Control, GC4419, or GC4404 groups.

To determine if catalytic cycling of the pentaazamacrocycle is required for the enhancement of AscH^−^ toxicity, we combined AscH^−^ with GC4404, a Mn(II)-pentaazamacrocyclic derivative that is devoid of SOD activity. GC4404 does not form the Mn(III)-intermediate, which has been suggested to be reduced by AscH^−^ [18,21]. Media were replaced, and H1299 lung cancer cells were treated with GC4419 or GC4404 for 1 h prior to and during the 1 h AscH^−^ treatment. As predicted, GC4419 enhanced the toxicity of AscH^−^ while GC4404 had no effect on AscH^−^ toxicity (Figure 4). These results further support the hypothesis that catalytic cycling of the pentaazamacrocycle is required for the enhancement of AscH^−^ toxicity.

### 3.5. GC/AscH^−^ Treatment Increases Cancer Cell Response to Ionizing Radiation

**Figure 5:**
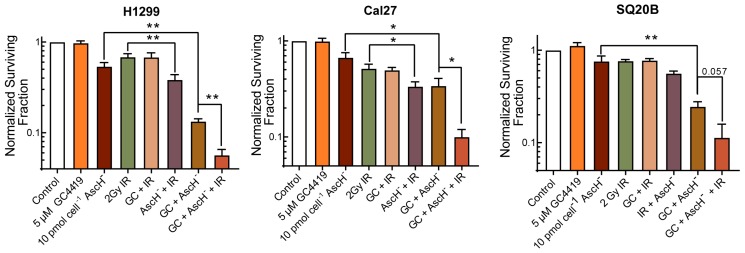
GC/AscH^−^ treatment sensitizes cancer cells to IR as seen by clonogenic assay. H1299 lung cancer cells and Cal27 and SQ20B head and neck cancer cells were treated with GC4419 24 h before and AscH^−^ 1 h before exposure to IR. Errors represent ± SEM, *n* = 3–4 with at least two cloning dishes per treatment. * *p* < 0.05, ** *p* < 0.01, by unpaired *t*-test. Normalized to Control.

Patients being treated with GC4419 in clinical trials to date (i.e., NCT02508389) all received radiation treatment; H_2_O_2_ is known to increase tumor response to radiation [5,31]. Thus, combining AscH^−^ and GC4419 with radiation has the potential to improve tumor therapy outcomes. To investigate the ability of GC/AscH^−^ to enhance the response of cancer cells to ionizing radiation (IR), cancer cells were exposed to GC4419 for 24 h and AscH^−^ for 1 h prior to exposure to IR. Interestingly, the treatment with GC/AscH^−^ prior to 2 Gy IR resulted in cancer cell killing that was at least additive and significantly exceeded that seen with either agent alone in two cell lines, with a third trending toward significance (Figure 5).

## 4. Discussion

We have shown that the Mn(II)-pentaazamacrocyclic SOD mimetic GC4419 increases the rate of oxygen consumption, [Asc^•−^]_ss_, and H_2_O_2_ production in complete media containing pharmacological levels of AscH^−^. When applied to cells, the combination of GC4419/AscH^−^ results in increased clonogenic cancer cell killing that is dependent on H_2_O_2_ and the catalytic activity of the pentaazamacrocycle. Another exciting finding is that the combination of GC4419 and AscH^−^ sensitizes cancer cells to IR.

Understanding the similarities and differences of reaction mechanisms of various classes of SOD mimetics with AscH^−^ is of potential importance for the clinical implementation of SOD mimetics in combination with AscH^−^. In addition, this understanding will be critical for the development of novel or structurally-related compounds that combine with AscH^−^ to enhance cancer cell toxicity. For the Mn(III)-porphyrins possessing SOD mimetic activity, it has been proposed that the Mn(III) is reduced by AscH^−^, yielding Mn(II) and Asc^•−^. The Mn(II) then reduces O_2_ to O_2_^•−^ [11].

$$\mathrm{Mn}\left(\mathrm{III}\right)+{\mathrm{AscH}}^{-}\to \mathrm{Mn}\left(\mathrm{II}\right)+{\mathrm{Asc}}^{•-}$$

$$\mathrm{Mn}\left(\mathrm{II}\right)+O_{2}\to \mathrm{Mn}\left(\mathrm{III}\right)+{O_{2}}^{•-}$$

Because the Mn(III)-porphyrin simply serves as a catalyst, the net reaction is:

$$O_{2}+{\mathrm{AscH}}^{-}\to {O_{2}}^{•-}+{\mathrm{Asc}}^{•-}$$

The O_2_^•−^ then dismutes spontaneously or through the action of the Mn(III)-porphyrin as an SOD mimetic to yield H_2_O_2_. Because catalase overexpression has been shown to completely inhibit killing, this H_2_O_2_ is principally responsible for the toxicity of AscH^−^ [10,11].

$${O_{2}}^{•-}+{O_{2}}^{•-}+2H^{+}\to H_{2}O_{2}+O_{2}$$

In the case of Mn(II)-pentaazamacrocyclic mimetics, we propose that the O_2_^•−^ or HO_2_^•^ necessary to initiate the oxidation of GC4419 is primarily produced by reactions between AscH^−^ and redox-active transition metals in cell culture media [32,33]. For example,

$${\mathrm{AscH}}^{-}+{\left(\mathrm{Cu}/\mathrm{Fe}\right)}^{n}\to {\mathrm{Asc}}^{•-}+{\left(\mathrm{Cu}/\mathrm{Fe}\right)}^{n-1}$$

$${\left(\mathrm{Cu}/\mathrm{Fe}\right)}^{n-1}+O_{2}\to {O_{2}}^{•-}+{\left(\mathrm{Cu}/\mathrm{Fe}\right)}^{n}$$

$${O_{2}}^{•-}+H^{+}↔{{\mathrm{HO}}_{2}}^{•}$$

As previously described, the Mn(II) of the pentaazamacrocycle is oxidized by two competing pathways [34,35]. In the first, Mn(II)OH_2_ reacts via an outer-sphere pathway with HO_2_^•^ to yield a Mn(III)OH intermediate and H_2_O_2_.

$$\mathrm{Mn}\left(\mathrm{II}\right){\mathrm{OH}}_{2}+{{\mathrm{HO}}_{2}}^{•}\to \mathrm{Mn}\left(\mathrm{III}\right)\mathrm{OH}+H_{2}O_{2}$$

We propose that the Mn(III)OH intermediate can then be reduced by hydrogen atom transfer from AscH^−^, yielding H_2_O, Mn(II), and Asc^•−^.

$$\mathrm{Mn}\left(\mathrm{III}\right)\mathrm{OH}+{\mathrm{AscH}}^{-}\to \mathrm{Mn}\left(\mathrm{II}\right)+H_{2}O+{\mathrm{Asc}}^{•-}$$

Removing the Mn(II)-pentaazamacrocycle from the equation, because it serves as a catalyst, the net reaction is:

$${{\mathrm{HO}}_{2}}^{•}+{\mathrm{AscH}}^{-}\to {\mathrm{Asc}}^{•-}+H_{2}O_{2}$$

The second mechanism is in-line with the conclusion that GC4419 can act as an AscH^−^/O_2_^•−^ oxidoreductase by catalyzing the transfer of an electron from AscH^−^ to O_2_^•−^, yielding H_2_O_2_ and Asc^•−^ [21]. In this reaction, the Mn(II) intermediate is complexed by O_2_^•−^ and oxidation is initiated following protonation of the bound O_2_^•−^.

$$\mathrm{Mn}\left(\mathrm{II}\right)+{O_{2}}^{•-}+H^{+}\to \mathrm{Mn}\left(\mathrm{III}\right)O_{2}H$$

We propose that the Mn(III)O_2_H intermediate is also then reduced by a hydrogen atom transfer from AscH^−^, yielding H_2_O_2_ and Asc^•−^.

$$\mathrm{Mn}\left(\mathrm{III}\right)O_{2}H+{\mathrm{AscH}}^{-}\to \mathrm{Mn}\left(\mathrm{II}\right)+H_{2}O_{2}+{\mathrm{Asc}}^{•-}$$

Removing the Mn(II)-pentaazamacrocycle complex from the equation, because it serves as a catalyst, the net reaction is:

$${O_{2}}^{•-}+H^{+}+{\mathrm{AscH}}^{-}\to H_{2}O_{2}+{\mathrm{Asc}}^{•-}$$

Several findings in the present study support these conclusions:

(1) the reduction of the Mn(III) intermediate by AscH^−^ is thermodynamically favorable, as the reduction potential of GC4419 Mn(III)/Mn(II) (+525 mV, acetonitrile) is considerably greater than Asc^•−^/AscH^−^ (+282 mV, aqueous) [32,36];
(2) Native CuZnSOD does not enhance cancer cell toxicity or H_2_O_2_ production when combined with AscH^−^, supporting the hypothesis that the SOD activity of GC4419 is not responsible for increased AscH^−^ toxicity;
(3) Addition of GC4419 to solutions containing AscH^−^ results in a significant increase in [Asc^•−^]_ss_, supporting the hypothesis that addition of GC4419 increases the one-electron oxidation of AscH^−^;
(4) The structurally-similar Mn(II)-pentaazamacrocycle complex, GC4404, which does not form the Mn(III)-intermediate which has been suggested to be reduced by AscH^−^ [35], does not enhance AscH^−^ cancer cell toxicity, strongly supporting the hypothesis that catalytic cycling of the pentaazamacrocycle is required for the enhancement of AscH^−^ toxicity; and
(5) GC4419 enhances H_2_O_2_ production when combined with AscH^−^, and the cancer cell toxicity of GC/AscH^−^ is completely inhibited by catalase overexpression, demonstrating that H_2_O_2_ is responsible for the toxicity of GC/AscH^−^.

## 5. Conclusions

Thus, our findings support the conclusion that, in the presence of mM levels of AscH^−^ in complete media, GC4419 can function as an AscH^−^/O_2_^•−^ oxidoreductase catalyst [21], killing human lung and head and neck cancer cells by a mechanism involving the formation of H_2_O_2_. However, the role of the two competing pathways of oxidation and how this relates to the potential AscH^−^/O_2_^•−^ oxidoreductase activity of GC4419 requires further investigation. Future studies will also investigate the utility of GC/AscH^−^ in enhancing the response of cancer cells and tumors to radiation and chemotherapy in vivo as well as examine the ability of other pentaazamacrocyclic compounds to enhance AscH^−^ cancer cell toxicity.

## 6. Patents

Authors Collin D. Heer, Robert A. Beardsley, Dennis P. Riley, and Douglas R. Spitz and have submitted one or more patent applications covering some of the aspects of these discoveries.
